# The Biological Basis of Mathematical Beauty

**DOI:** 10.3389/fnhum.2018.00467

**Published:** 2018-11-30

**Authors:** Semir Zeki, Oliver Y. Chén, John Paul Romaya

**Affiliations:** ^1^Laboratory of Neurobiology, University College London, London, United Kingdom; ^2^Department of Psychology, Yale University, New Haven, CT, United States

**Keywords:** mathematical beauty, biological beauty, artifactual beauty, deductive logic, neuroesthetics

## Abstract

Our past studies have led us to divide sensory experiences, including aesthetic ones derived from sensory sources, into two broad categories: biological and artifactual. The aesthetic experience of biological beauty is dictated by inherited brain concepts, which are resistant to change even in spite of extensive experience. The experience of artifactual beauty on the other hand is determined by post-natally acquired concepts, which are modifiable throughout life by exposure to different experiences (Zeki, 2009; Zeki and Chén, 2016). Hence, in terms of aesthetic rating, biological beauty (in which we include the experience of beautiful faces or human bodies) is characterized by less variability between individuals belonging to different ethnic origins and cultural backgrounds or the same individual at different times. Artifactual beauty (in which we include the aesthetic experience of human artifacts, such as buildings and cars) is characterized by greater variability between individuals belonging to different ethnic and cultural groupings and by the same individual at different times. In this paper, we present results to show that the experience of mathematical beauty (Zeki et al., 2014), even though it constitutes an extreme example of beauty that is dependent upon (mathematical) culture and learning, is consistent with one of the characteristics of the biological categories, namely a lesser variability in terms of the aesthetic ratings given to mathematical formulae experienced as beautiful.

## Introduction

In an earlier study (Zeki et al., 2014), we reported that the experience of mathematical beauty (by mathematicians) correlated with activity in field A1 of the medial orbito-frontal cortex (A1mOFC); moreover, the intensity of activity there was parametrically related to the declared intensity of the beauty experienced by the mathematicians when viewing the mathematical formulae. This was a somewhat surprising result, at least to us. The experience of mathematical beauty is derived from a highly intellectual, cognitive source; it is indeed this very source and its preoccupation with eternal and immutable truths that led Plato (1961a,b) to consider mathematical beauty as the highest form of beauty. It is also the most extreme example of aesthetic experience that is dependent upon culture and learning. Unlike visual or musical beauty, *only those versed in mathematics* can experience the beauty of mathematical formulations. And yet the experience of mathematical beauty correlates with activity in the same part of the emotional brain as the experience of beauty derived from sensory sources, such as the visual or the musical (Ishizu and Zeki, 2011). This naturally leads one to enquire further into the nature and classification of mathematical beauty. We have in the past classified sensory experiences in general, including aesthetic ones, into two broad categories, biological and artifactual (Zeki, 2009; Zeki and Chén, 2016); the former are interfaced through inherited brain concepts and are less dependent on culture and learning than the latter, which are interfaced through acquired concepts that are modifiable throughout post-natal life through exposure to new experiences. In particular, we posited that experiences regulated by inherited biological concepts are more widely shared and less variable than ones regulated by acquired concepts. There is some evidence to support this classification (Chén and Zeki, 2011), which, in outline, has also been confirmed by recent results on the experience of beauty derived from sensory visual sources (Vessel et al., 2018). The question that we address here is which category mathematical beauty belongs to, given that it is an experience that is heavily dependent upon learning and culture on the one hand but which requires unanimity for its validity on the other.

We therefore wanted to add to our previous study of the experience of mathematical beauty by analyzing our results further, with the following questions in mind: what was the degree of variability in the ratings given to equations that had been rated as beautiful and did that variability differ in any significant way from the variability in the “non-beautiful” ratings that had been assigned to other equations? Our only hypothesis in this regard was that, if mathematical beauty belongs to the biological category, then there should be significantly less variability among equations given high ratings than among others. We indeed found this to be the case, which reinforced our view that mathematical beauty belongs to the category of biological beauty, for reasons which we have explored before (Zeki et al., 2014) and explore more fully here in the Discussion section.

## Material and methods

A full description of the subjects and methods used to rate mathematical equations is given in Zeki et al. (2014), where all the 60 mathematical formulae used in the study are also tabulated. The ratings used appear in Data Sheet 3 of the supplemental data in Table 1 (pre-scan beauty ratings) and 6 (post-scan understanding ratings) of Zeki et al. (2014).

In brief, 15 mathematicians (three females, in the age range of 22–32 years) and all of them post-graduate students or post-doctoral fellows in mathematics took part in the study. Each was given the 60 mathematical equations to study at leisure and rate according to the aesthetic experience aroused in them on a scale of −5 (ugly) to +5 (beautiful). Subsequent to a brain scanning experiment, to determine the brain areas in which activity correlates with the experience of mathematical beauty (the results of which are reported in Zeki et al., 2014), each subject was asked to report their level of understanding of each equation on a scale of 0 (no understanding) to 3 (profound understanding) and to report their emotional reactions to the equations. In this paper we use the pre-scan beauty ratings from our earlier study (Zeki et al., 2014); these were scored on an 11-point scale of −5 to +5, unlike the scan-time beauty ratings, which were rated on a 3-point scale of −1/0/+1. We also use the post-scan understanding ratings (0–3).

As for comparison, we asked 12 controls (i.e., non-mathematicians) to give beauty and understanding ratings to the same equations, exactly as for the mathematicians (see Zeki et al., 2014). The majority gave an understanding rating of 0 to most equations, with some giving positive beauty ratings to a minority of the equations. One subject did not give beauty ratings to any of the equations and rated all 0's for understanding, effectively leaving us with 11 subjects as controls. Overall, of the 720 equations distributed over the non-mathematical subjects, 645 (89.6%) were given a 0 rating (no understanding), 49 (6.8%) were given a rating of 1 (vague understanding) and the remainder were rated as 2 (good understanding) or 3 (profound comprehension). The majority (9 out of 12) gave a negative response to the question: “*When you consider a particularly beautiful equation, do you experience an emotional response?*” Given this, we hypothesized that, when such non-mathematical subjects gave a positive beauty rating to the equations, they were doing so on a formal basis; that is to say, on how attractive the form of the equations was to them. Even so, the results in Figure 2 show that there was no uniformity among controls for rating formulae according to formal qualities (see also Table 1 in the Discussion).

**Table 1:** The five equations (out of 60) given the top ratings (on a scale of −5 to +5) are shown in the upper section while those given the lowest ratings are shown below.

**#**	**Equation**	**Description**	**Mean rating**
1	1+*e*^*iπ*^ = 0	Euler's identity links five fundamental mathematical constants with three basic arithmetic operations each occurring once.	3.6000
2	cos^2^θ +sin^2^θ = 1	The Pythagorean identity, which states that for any angle, the square of the sine plus the square of the cosine is 1.	3.2667
54	$\frac{\partial u}{\partial x}=\frac{\partial v}{\partial y},\;\frac{\partial u}{\partial y}=-\frac{\partial v}{\partial x}$	Cauchy-Riemann equations are a system of two partial differential equations which must be satisfied if a complex function is complex differentiable.	3.1333
5	*e*^*ix*^ = cos*x*+ *i*sin*x*	Identity between exponential and trigonometric functions derivable from Euler's formula for complex analysis.	3.0000
6	$\overset{\infty }{\underset{-\infty }{\int }}e^{-x^{2}}dx=\;\sqrt{\pi }$	Definite Gaussian integral-ubiquitous in mathematical physics.	2.9333
. . .			
39	|∅| = 0	The cardinality of the empty set is zero.	−0.4000
51	*U*^*C*^ = ∅	The complement of the universal set is the empty set.	−0.5333
15	1729 = 1^3^+ 12^3^ = 9^3^+ 10^3^	The smallest number expressible as the sum of two cubes in two different ways.	−1.1333
28	3^2^+4^2^ = 5^2^	Pythagoras' theorem for a 3:4:5 triangle.	−1.1333
14	$\frac{1}{\pi }=\frac{2\sqrt{2}}{9801}\overset{\infty }{\underset{k=0}{\sum }}\frac{\left(4k\right)!\left(1,103+26,390k\right)}{{\left(k!\right)}^{4}\;396^{4k}}$	Equation expressing the inverse value of π as an infinite sum.	−1.8000

## Results

### Primary finding

In our statistical analyses of the results we use the following notations:

Let *r*_*ij*_ denote the **beauty rating** that the *i*^*th*^ subject gives to the *j*^*th*^ formula; let *u*_*ij*_ denote individual *i*'s **understanding** of the *j*^*th*^ formula; let *N* denote the total number of subjects; let $x_{j}:=\overset{N}{\underset{i=1}{\sum }}r_{ij}/N$ and $y_{j}:=\sqrt{\overset{N}{\underset{i=1}{\sum }}{\left(r_{ij}-x_{j}\right)}^{2}/\left(N-1\right)}$ be the mean beauty rating (**m-BR**) and standard deviation of beauty ratings (**sd-BR**), given to the *j*^*th*^ formula across subjects, respectively; let $\mu_{j}:=\overset{N}{\underset{i=1}{\sum }}u_{ij}/N$ and $\sigma_{j}:=\sqrt{\overset{N}{\underset{i=1}{\sum }}{\left(u_{ij}-\mu_{j}\right)}^{2}/\left(N-1\right)}$ be the mean understanding rating of the formula (**m-UR**) and standard deviation of the understanding rating (**sd-UR**), given to the *j*^*th*^ formula across subjects, respectively.

We undertook the following statistical analyses on the ratings.

**Figure 1:**
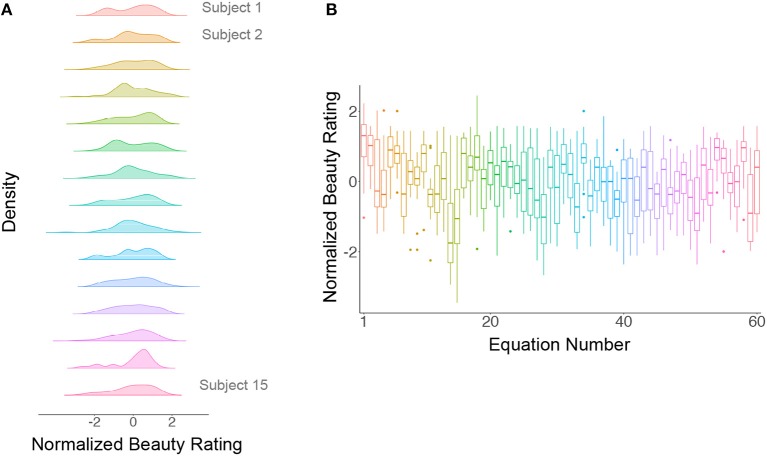
**(A)** Normalized ratings against subjects. The distribution of the normalized ratings for each subject is approximately *Gaussian* with a mean of 0 and a standard deviation of 1. **(B)** Normalized ratings against equations. The ratings for each equation are represented by a box plot; the horizontal line within each box plot represents the median of the ratings and the vertical lines represents the first and the last quartile of the ratings, respectively, while the box itself represents the middle 50% of the ratings. Dots represent outlier ratings.

**Figure 2:**
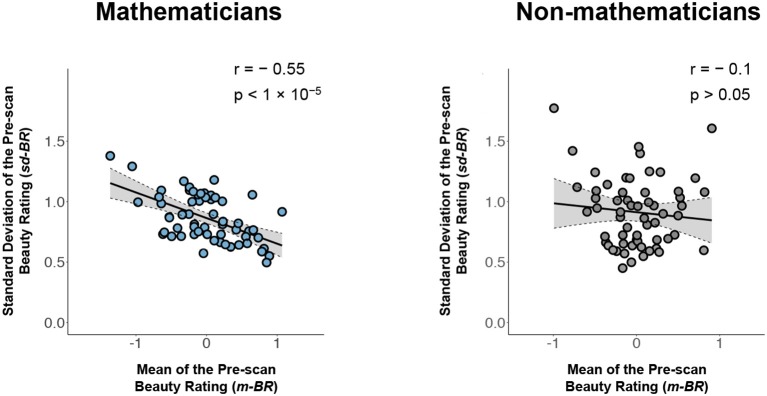
Left: A plot of the mean pre-scan beauty rating (m-BR) for each equation against the standard deviation (sd-BR) of the ratings given to each equation for mathematicians (Pearson *r*: −0.55, *p* < 10^−5^). Right: The same plot for non-mathematicians (Pearson *r*: −0.10, *p* > 0.05). Each circle corresponds to one equation; its value on the horizontal line indicates the average beauty ratings of the equation across all subjects and its value on the vertical line indicates the standard deviation of the beauty ratings. Gray area indicates the 95% confidence band for best-fit line.

1. We first normalized the beauty rating scores for each subject, following which the ratings from each subject were centered at 0 with a standard deviation of 1. The intra-class (between subject) correlation coefficient (ICC) for ratings across subjects became −0.02, indicating that there was no tendency for subjects to systematically give all equations higher or lower ratings (see Figure 1A). In other words, the source of variability in beauty ratings (see Figure 1B) must be either specific to the equations, or to another linked source (e.g., variability in the understanding of the equations), but unlikely to be due to between-subject variability. Similarly, we normalized the understanding scores for each subject, to obtain similar scales for the beauty and the understanding ratings; this gave an ICC = −0.02.
2. We calculated the m-BR and the sd-BR (i.e., the mean and standard deviation of the normalized beauty ratings) as well as the m-UR and sd-UR (mean and standard deviation of the normalized understanding ratings), for each formula across subjects. This gave 60 m-BR values with 60 corresponding sd-BR values, and 60 m-UR values with 60 corresponding sd-UR values. Although the range for beauty ratings was from −5 to 5, and that for understanding was from 0 to 3, the ranges for m-BR and m-UR, after normalization, are (−1.36, 1.07) and (−1.31, 1.08), respectively. Therefore, we removed, through normalization, confounding effects that can be caused by a difference in the ratings' original scales. For simplicity, data analyses were all conducted using normalized data, and we therefore omit the term normalized below.
3. We plotted the m-BR values against the sd-BR values for both mathematicians and non-mathematicians (see Figure 2). The graph for the mathematicians (left) has a pronounced negative trend, showing that there was generally a lower standard deviation for formulae rated as beautiful compared to ones not rated as beautiful (Pearson *r*: −0.55, *p* < 10^−5^). In simpler terms, there was a higher consensus among our sample of 15 subjects regarding beautiful equations than about the not beautiful ones since, unlike the equations rated as beautiful, there was greater variability for those rated as not beautiful. This is the primary finding reported here.

In contrast, the graph relating the m-BR to the sd-BR for the controls (non-mathematicians) (right in Figure 2) is nearly flat and shows no significant correlation between the two ratings. Therefore, unless otherwise specified, in the following we focus on inquiring into the relationship of m-BR, sd-BR, and potential confounds only among mathematicians.

### Possible confounds due to understanding rating

**Figure 3:**
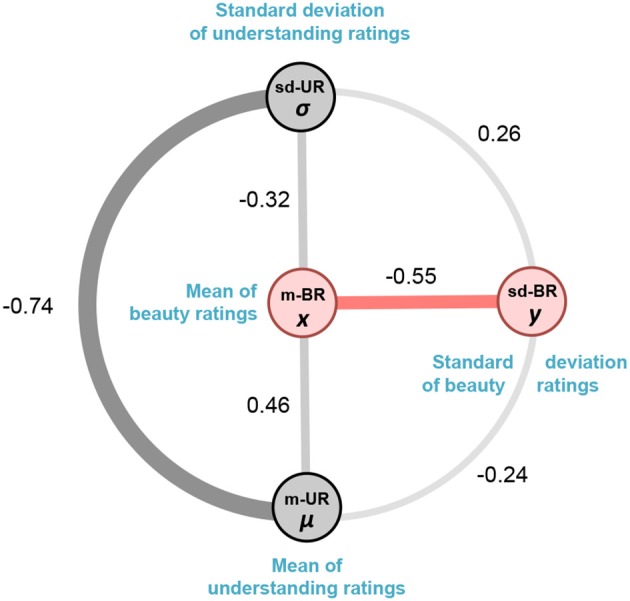
A schematic representation of the association between mean beauty ratings (m-BR), mean understanding ratings (m-UR), the standard deviation of understanding ratings (sd-UR) and standard deviations of beauty ratings (sd-BR). The thickness of the connecting lines denotes the pairwise Pearson correlations between them. We show below that although there is an association between m-BR and m-UR, there is no association between m-UR and sd-BR. Moreover, adding m-UR to m-BR does not improve the established association between m-BR and sd-BR.

Although there is a significant relationship between m-BR and sd-BR, there also exists a possible confound since we know of (and might reasonably expect) a positive correlation between the mean beauty ratings and the mean understanding ratings of the equations. Thus, the relationship between m-BR and sd-BR might primarily be due to the understanding rating rather than the beauty rating. Figure 3 depicts the correlations between the beauty and understanding ratings and other confounds. In the following, we conduct further analyses to rule out any influence of the understanding ratings on the beauty ratings.

**Figure 4:**
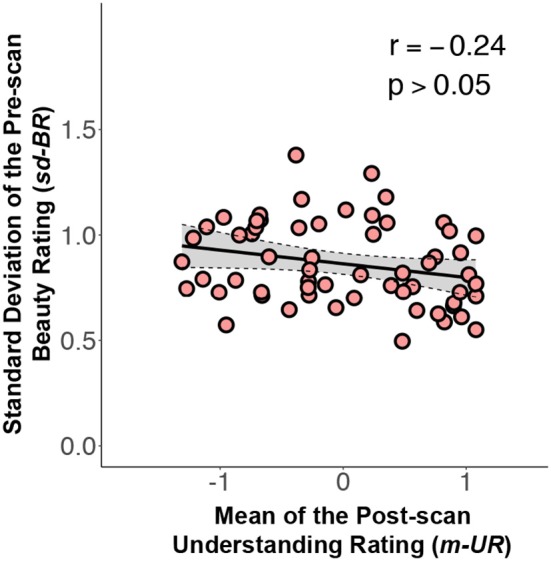
A graph relating the standard deviation of the pre-scan beauty ratings (sd-BR) against mean understanding ratings (m-UR) for each equation (Pearson *r*: −0.24, *p* > 0.05). Conventions as in Figure 2.

1. We investigate first the linear relationship between the m-BR and the sd-BR across equations. Formally, consider the equation
  (1) $$\begin{array}{r} y_{j}=\beta_{0}+\beta_{1}x_{j}+\varepsilon_{j},\;1\leq j\leq 60 \end{array}$$
  where *x*_*j*_ and *y*_*j*_ refer to the m-BR and the sd-BR of the *j*^*th*^ equation, β_0_ and β_1_ are parameters for the intercept and slope, and ε_*j*_ indicates the residual term (i.e., the information not explained). Our data shows that the estimate for β_1_, or ${\hat{\beta }}_{1}=$ −0.21 (*p* < 10^−5^). In other words, if a formula is on average rated one point higher than a second formula, the standard deviation of the ratings across subjects (which quantifies the disagreement among subjects) for the former formula is 0.21 units less than the standard deviation of the ratings for the latter formula. More simply stated, this means that where there is a higher beauty rating for a mathematical formula, there is less variation in the rating among individuals.
2. Although a low sd-BR is significantly associated with a high m-BR (the more beautifully rated formulae have smaller standard deviations), it remained possible that these are confounded by subjects' understanding of the mathematical formulae. To check for confounds, we reran Model (1), where the regressor *x*_*j*_ (the m-BR of formula *j*) was replaced with μ_*j*_ (the m-UR of formula *j*) (see Figure 4). Our data showed that the m-UR is not significantly associated with the sd-BR (Pearson *r*: −0.24, *p* > 0.05).
3. So far, we have shown that the m-UR was not significantly associated with the sd-BR; the possibility remains, however, that the m-UR and the m-BR may *jointly* affect the sd-BR. We therefore ran tests to learn if adding the m-UR to the m-BR would give a better account of the variability in sd-BR. Formally, using an F-test, we compared Model (1) with the following model
  (2) $$\begin{array}{r} y_{j}=\beta_{0}+\beta_{1}x_{j}+\beta_{2}\mu_{j}+e_{j},\;1\leq j\leq 60 \end{array}$$
  where μ_*j*_ denotes the m-UR of the *j*^*th*^ formula, and *e*_*j*_ indicates the residual term. The *F*-test can be used for determining whether a “more complicated” model with additional parameters [e.g., Model (2)] is *significantly* better than a baseline model [e.g., Model (1)].
  The *F*-test statistic is defined as $F=\frac{\left(RSS_{1}-RSS_{2}\right)/\left(df_{1}-df_{2}\right)}{RSS_{2}/df_{2}}$, where *RSS*_1_ and *RSS*_2_ denote residual sums of squares for Models (1) and (2), respectively, and *df*_1_ and *df*_2_ denote degrees of freedom (i.e., number of data points minus number of parameters) for Models (1) and (2), respectively (Fisher, 1925; Ott and Michael, 1980; Bickel and Doksum, 2000).
  Using this test, we show that adding the m-UR (mean understanding rating, μ) in Model (2) does not significantly reduce prediction errors in Model (1) (*F* = 0.04, *p* = 0.84) and therefore does not add additional information to the established association between the sd-BR (*y*) and the m-BR (*x*) in Model (1).
4. Taken together, our analyses show that high mean beauty rating of formulas in a population is significantly (negatively) associated with the standard deviation of the beauty ratings. Specifically, one unit increase of mean rating leads to −0.21 units decrease of standard deviation of a formula. Further, such association is neither due to, nor can be further explained by, one's understanding of the formulas, suggesting that there is a unified aesthetical appreciation of mathematics among individuals, and that such aesthetic appreciation is separate from one's understanding of the mathematical formulae.

### Possible ceiling effect

Since the beauty rating scales were from −5 to 5, it is possible that there was a ceiling effect for those equations that were rated either −5 or 5. In other words, were the scale changed to −10 to 10, some of those equations that were rated 5 and −5 on our scale of −5 to 5 may have been rated lower than −5 or higher than 5. If so, then the ceiling effect could have potentially reduced the variance of the highly (or lowly) rated equations, thus confounding our results. Although normalization reduces the ceiling effect, it could still be argued that the results would be different (and possibly insignificant) were the ratings on a different scale, even with normalization. We therefore conducted simulation studies to learn whether such a ceiling effect, even if it exists in our studies, would modify our conclusion.

**Figure 5:**
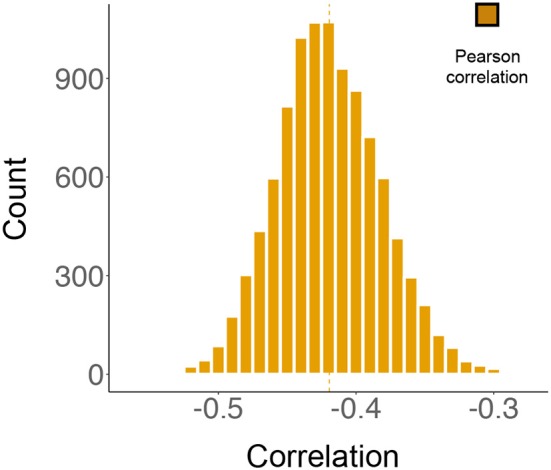
The results from simulation studies. The histogram represents 10,000 correlations between the mean beauty ratings (m-BR) and the standard deviation of the beauty ratings (sd-BR), analyzed by Pearson's test. During each simulation, non-negative integers generated from a Poisson distribution were added to (or subtracted from) the original ratings of 5 (or −5). The 95% confidence interval for the distribution is (−0.420, −0.419).

1. The likelihood of a ceiling effect for ratings between −4 and 4 is low, because one can always choose a higher (i.e., 5) or lower (i.e., −5) rating. Thus, we focused on addressing the potential ceiling effect with regards to ratings of 5 and −5. The first set of simulations was conducted as follows: for any equation that was rated 5, we simulated a positive integer and added it to 5. Similarly, for any equation that was rated −5, we simulated another positive integer and subtracted it from −5. Each equation-specific integer *K* was simulated from a Poisson distribution with $\mathbb{P}\left(K=k\right)=e^{-\lambda }\frac{\lambda^{k}}{k!}$ , where λ = 1. We chose a Poisson distribution because it generates non-negative integers, so the resulting numbers are still valid integer ratings; we set parameter λ = 1 because the data thus simulated approximates a scenario with a relatively serious ceiling effect, i.e., it simulates a (worst case) situation where there is a 63.15% chance of having a ceiling effect. Where there was a ceiling effect with this procedure we added (or subtracted) 1 to 5 points to a rating of 5 (or from a rating of −5) according to the above probability distribution. There was a 99.94% chance that the added or subtracted integers were less than, or equal, to 5, thereby placing the majority of the final ratings between −10 and 10; there was a 0.06% chance that the added (or subtracted) integers were >5, simulating a real world scenario where a subject could possibly have wanted to rate a formula beyond the range of 10 to −10, that is to say well outside the prescribed limits of −5 to 5.
2. We repeated the above simulation study 10,000 times, in each of which different randomly simulated non-negative integers were added to (or subtracted from) 5's (or −5's). The 95% confidence interval for the 10,000 Pearson correlations between the simulated sd-BR and m-BR ranged from −0.420 to −0.419, and the 95% confidence interval for the corresponding *p*-values fell between 1.5 × 10^−3^ and 1.6 × 10^−3^ (see Figure 5).
3. Taken together, even if there was a ceiling effect, the significant association between sd-BR and m-BR still exists; this conclusion is based on extensive simulations, where serious ceiling effects were considered for the original rating data.

## Discussion

That the experience of mathematical beauty (Zeki et al., 2014) correlates with activity in the same part of the emotional brain, namely A1mOFC, as the experience of beauty derived from other sources, including sensory and moral ones (Ishizu and Zeki, 2011), raises the question of how to classify mathematical beauty.

We have in the past suggested that the classification of experiences, ranging from ordinary sensory ones, such as that of color, to aesthetic experiences, such as that of beauty, can be subdivided into two broad categories (Zeki, 2009; Zeki and Chén, 2016), a categorization that finds confirmation in recent psychophysical studies (Vessel et al., 2018). At one end are experiences belonging to the *biological* category: these have a biologically inherited brain concept as a basis (Zeki, 2009). Such biological concepts lead to experiences that are shared universally by all ethnic groups and are independent of learning. Moreover, they are not easily modifiable by experiment or repeated exposure to a variant that departs significantly from the inherited concept, at least within the limits tested (see Chén and Zeki, 2011). This entitles a subject having an aesthetic experience which belongs in the biological category and determined by an inherited brain concept to suppose that the experience is similar to the one that others would have in similar circumstances and that it has, therefore, universal validity and assent (see also Zeki and Chén, 2016). A prime example of this is in the experience of color categories. The colors of objects and surfaces remain constant in spite of wide fluctuations in the wavelength-energy composition of the light reflected off them (Land, 1974). This phenomenon is generally referred to as color constancy although we much prefer the term constant color categories (see Zeki et al., 2017). This is because, although the color category does not change with such fluctuations, the exact hue (shade of color) of a patch belonging to a given color category will do so. The generation of constant color categories is due, we suppose, to an inherited brain program which compares the wavelength-energy composition of light coming from one patch with that coming from surrounding patches, thus generating ratios between the two for every waveband (Land, 1974), with the ratios remaining constant in spite of significant changes in the amounts of light of different wavebands reflected from the viewed patch and its surrounds. In fact, psychophysical experiments that we have undertaken (in preparation) demonstrate that there is very little variability among humans of different ethnic and cultural backgrounds when asked to assign the color of patches (which are reflecting light of the same wavelength-energy composition) to a standard set of colored chips. This is because, due to an inherited brain program for generating constant color categories, the patches maintain their color categories even with wide variations in the wavelength-energy composition of the light reflected from them. Hence humans can (and do) suppose that others have a similar color experience to them and that their experience has, therefore, universal, assent.^1^

At the other end are experiences determined by acquired brain concepts, examples being that of man-made artifacts consisting of a variety of manufactured goods. The concept underlying these experiences are acquired post-natally and are modifiable throughout post-natal life (Zeki, 2009), which can be demonstrated experimentally (Chén and Zeki, 2011). Because they are based on individual experience and experimentation, the experiencing subject cannot assume that others will have the same experience. As an example, someone who is brought up in a particular environment, say a Western one, cannot assume that another human from a different cultural environment will find the same satisfaction in manufactured goods from Western culture (in this category, we include such items as planes, cars, forks and knives, *etc*.). Moreover, since the brain concept itself is acquired post-natally and changes with new experiences, one cannot even assume that an aesthetic judgment on the architectural merit of a building made today will be the same as the one made in the past or that will be made in the future (Zeki and Chén, 2016).

### The categorization of mathematical beauty

This naturally raises the awkward question of whether the experience of mathematical beauty belongs in the biological or the artifactual category.

The experience of mathematical beauty is perhaps the most extreme aesthetic experience that is dependent upon culture and learning; those not versed in the language of mathematics cannot experience the beauty of a mathematical formulation. And yet, once the language of mathematics is mastered, the same formulae can be experienced as beautiful by mathematicians belonging to different races and cultures. Indeed, Paul Dirac coined the term “the principle of mathematical beauty” (Farmelo, 2011) and made the beauty of a mathematical formula, rather than its simplicity, the ultimate guide to its truthfulness (Dirac, 1939). He was not alone; other mathematicians, such as Hermann Weyl and Paul Erdös, thought similarly.

In what does the beauty of a mathematical formula lie? We gave thought to the possibility that the beauty ratings given to our mathematical equations had “low-level” sensory sources, such as curvatures, the position and number of elements, symmetry, and so on. Although this remains a remote possibility, we discount it and Table 1 below shows why. In that Table, we show the five formulae given the highest ratings and the five given the lowest ratings. The spatial characteristics (e.g., size, height, number of elements, symbols or symmetry) of the equations are broadly equivalent and the possibility that such factors, rather than the cognitive beauty, played a role in the ratings seems unlikely to us. Moreover, if the ratings were made on the basis of formal qualities, one might have expected some unanimity among the non-mathematicians as well. But the graph relating m-BR to sd-BR in our control group of non-mathematicians (see Figure 2, right) shows no consistent relation between the various formal characteristics of the formulae given in Table 1 and the ratings. This reinforces our view that such characteristics probably played no role in the ratings, while acknowledging the possibility that they may yet be shown to play a role.

### The determinants of mathematical beauty

Perhaps the most forceful way of accounting for the experience of mathematical beauty, and the one nearest to our belief, comes from Immanuel Kant on the one hand and Bertrand Russell on the other. Kant's views are opaque and difficult to understand, and his use of the term “intuition” especially vague. For an interpretation of what constitutes mathematical beauty for Kant we rely on Breitenbach's (2015) discussion. For Kant, it seems, a mathematical formulation is beautiful if it “makes sense.” This, of course, raises the question of “makes sense” to what. We believe that at least part of the experienced beauty of a mathematical formulation lies in the fact that it adheres to the logical deductive system of the brain, which is similar in individuals of all races and cultures, and hence makes sense in terms of that logical deductive system. This is clearly stated by Russell (1920) in his *Introduction to Mathematical Philosophy*. Although he makes no reference to the brain, Russell implicitly equates mathematics with the brain's logical deductive system when he asks “*What is this subject, which may be called indifferently either mathematics or logic?*” because, to him, “*What can be known, in mathematics and by mathematical methods, is what can be deduced from pure logic*” since “*logic is the youth of mathematics and mathematics is the manhood of logic*.” Perhaps most significantly for our argument, to Russell “*Logical propositions are such as can be known a priori, without study of the actual world*.” In other words, logical propositions can be traced to inherited brain concepts.

The implication of the statements made by Russell and others quoted above can be taken to mean that there is a biological basis to mathematical logic and, by extension, a biological basis to the experience of mathematical beauty. Our results are not inconsistent with such a supposition; but we are anxious to emphasize that they are merely suggestive in that direction and that we cannot assert, through them alone, that mathematical beauty is incontrovertibly biological in nature. Rather, we believe that our work, reported here, opens a new and useful discourse on the roots of mathematical beauty and how it can be studied and quantified.

The logical deductive system of the brain, whatever its details, is inherited and is therefore similar in mathematicians belonging otherwise to different races and cultures. It is in this sense that mathematical beauty has its roots in a biologically inherited logical-deductive system that is similar for all brains. It is only by adhering to the rules of the brain's logical deductive system that a formulation can gain universal assent and be found beautiful. Any departure from that would mean that it has lost the universal agreement. Implicit in our argument is that the experience of mathematical beauty, being the result of the application of the brain's logical-deductive system, is a demonstration that the logical deductive system of mathematical brains, no matter what their cultural background may be, is the same. And since mathematical beauty, in our categorization, belongs to the biological category, it is not surprising that there is significantly less variability among mathematicians in rating mathematical equations as beautiful.

## Ethics statement

All subjects gave written informed consent in accordance with the Declaration of Helsinki. The protocol was approved by the University College London Ethics Committee for experiments with human participants.

## Author contributions

SZ designed the project, analyzed the results with JR and wrote the paper. JR ran the experiments and analyzed the results, and contributed to the statistical section. OC undertook the statistical analyses.

### Conflict of interest statement

The authors declare that the research was conducted in the absence of any commercial or financial relationships that could be construed as a potential conflict of interest.

## Footnotes

^1^When we speak of similar color experiences, we restrict ourselves to saying that different humans do not differ when assigning different colored patches, surfaces or objects to chips belonging to different color categories. In saying so, we do not address the vexed question of color qualia.
