# Corrigendum: The Biological Basis of Mathematical Beauty

**DOI:** 10.3389/fnhum.2019.00325

**Published:** 2019-09-20

**Authors:** Semir Zeki, Oliver Y. Chén, John Paul Romaya

**Affiliations:** ^1^Laboratory of Neurobiology, University College London, London, United Kingdom; ^2^Department of Psychology, Yale University, New Haven, CT, United States

**Keywords:** mathematical beauty, biological beauty, artifactual beauty, deductive logic, neuroesthetics

In the original article, there was an error. The square root symbol is missing from our description of the results.

A correction has been made to the **Results** section, subsection **Primary Finding**, paragraph two:

“Let *r*_*ij*_ denote the **beauty rating** that the *i*^*th*^ subject gives to the *j*^*th*^ formula; let *u*_*ij*_ denote individual *i*'s **understanding** of the *j*^*th*^ formula; let *N* denote the total number of subjects; let $x_{j}:=\overset{N}{\underset{i=1}{\sum }}r_{ij}/N$ and $y_{j}:=\sqrt{\overset{N}{\underset{i=1}{\sum }}{\left(r_{ij}-x_{j}\right)}^{2}/\left(N-1\right)}$ be the mean beauty rating (**m-BR**) and standard deviation of beauty ratings (**sd-BR**), given to the *j*^*th*^ formula across subjects, respectively; let $\mu_{j}:=\overset{N}{\underset{i=1}{\sum }}u_{ij}/N$ and $\sigma_{j}:=\sqrt{\overset{N}{\underset{i=1}{\sum }}{\left(u_{ij}-\mu_{j}\right)}^{2}/\left(N-1\right)}$ be the mean understanding rating of the formula (**m-UR**) and standard deviation of the understanding rating (**sd-UR**), given to the *j*^*th*^ formula across subjects, respectively.”

The authors apologize for this error and state that this does not change the scientific conclusions of the article in any way. The original article has been updated.

